# Poster Session I - A70 CANADIAN EXPERIENCE WITH ENDOSCOPIC ULTRASOUND-GUIDED GASTROJEJUNOSTOMY: A CASE SERIES

**DOI:** 10.1093/jcag/gwaf042.070

**Published:** 2026-02-13

**Authors:** A Dashti, C Roda, A Jain, I Gan, M Byrne, R Trasolini

**Affiliations:** Gastroenterology, University of British Columbia, Vancouver, BC, Canada; Gastroenterology, University of British Columbia, Vancouver, BC, Canada; Gastroenterology, University of British Columbia, Vancouver, BC, Canada; Gastroenterology, University of British Columbia, Vancouver, BC, Canada; Gastroenterology, University of British Columbia, Vancouver, BC, Canada; Gastroenterology, University of British Columbia, Vancouver, BC, Canada

## Abstract

**Background:**

Endoscopic ultrasound-guided gastrojejunostomy (EUS-GJ) using a lumen-apposing metal stent (LAMS) is a minimally invasive therapy for gastric outlet obstruction (GOO), offering durable symptom relief and faster recovery than surgical or enteral approaches. This study describes successful application of this novel technique in a Canadian setting.

**Aims:**

To evaluate procedural and clinical outcomes of EUS-GJ at a Canadian tertiary centre.

**Methods:**

A retrospective single-centre review was conducted at Vancouver General Hospital (Aug 2023–May 2025). All consecutive patients undergoing planned EUS-GJ for malignant or benign GOO were included. Demographic, procedural, and clinical data were collected. Technical success was defined as successful LAMS deployment, and clinical success as achieving a Gastric Outlet Obstruction Scoring System (GOOSS) of >1without recurrent obstruction within 30 days.

**Results:**

Twenty-four patients underwent attempted EUS-GJ (median age 66.5 years [IQR 61.2–73.5]; 46% female). Twenty-two (92%) had malignant obstruction, most commonly pancreatic adenocarcinoma (45%) and two benign indications including afferent limb syndrome and pyloric stenosis. Median ASA class was 3. The obstruction was most commonly duodenal (75%), followed by antral or pyloric (17%). Nearly half had prior enteral stenting or dilation, highlighting EUS-GJ as salvage therapy in refractory obstruction. All procedures were performed under general anesthesia using electrocautery-enhanced LAMS (15–20mm x10mm). Transgastric access was used in 22 (92%) and transbulbar in 2 (8%).

Technical success was achieved in 23/24 (95.8%). One procedure was aborted due to interposed ascites with successful deployment of a duodenal stent instead. Clinical success was achieved in 23/24 (95.8%). Median GOOSS improved from 0 [IQR 0–0.8] to 3 [IQR 2.3–3.0]. Median hospital stay was 5 days (IQR 1.8–10.2).

Two adverse events occurred: one aspiration requiring brief ICU stay, and one contained perforation adjacent to a pre-existing gastric stent managed non-operatively. No LAMS-related perforations, misdeployments, or surgical rescues occurred. Several patients resumed systemic therapy within weeks, reflecting rapid recovery and preserved performance status.

**Conclusions:**

EUS-GJ with LAMS achieved high technical and clinical success with minimal morbidity and short hospitalization in a medically complex Canadian cohort. These findings illustrate safe and effective integration of advanced therapeutic EUS in real-world tertiary practice and support EUS-GJ as a durable, minimally invasive option for palliation of GOO.

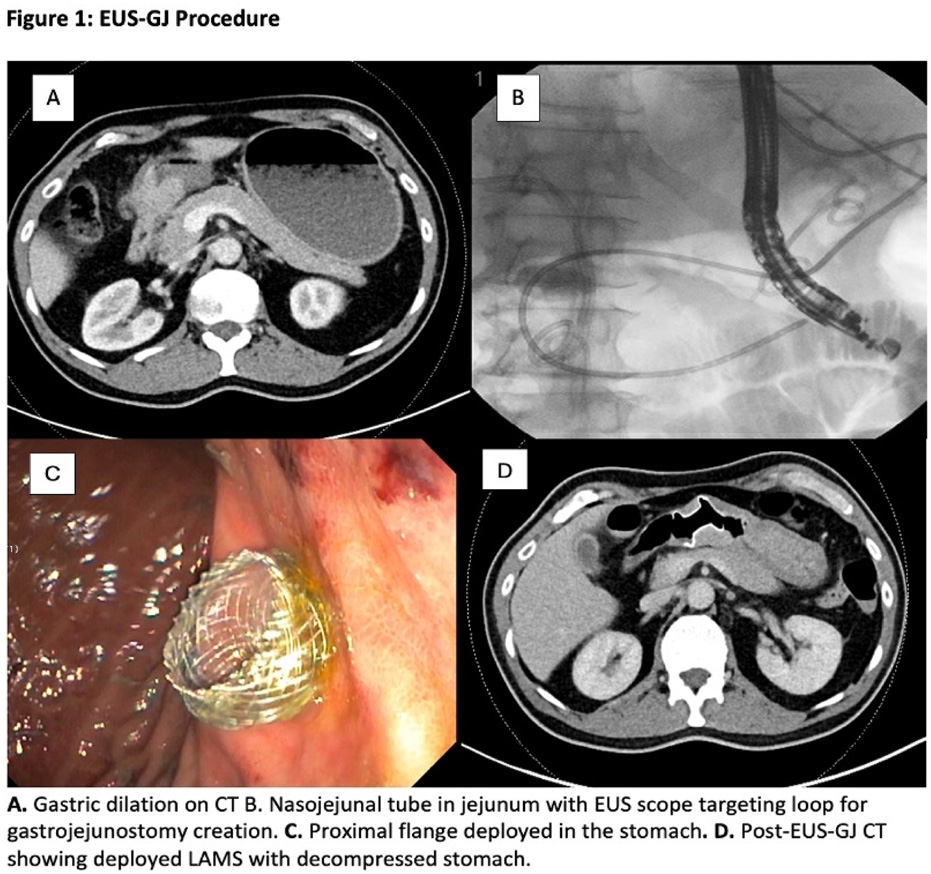

**Funding Agencies:**

None

